# New Sufficient Conditions for Hamiltonian Paths

**DOI:** 10.1155/2014/743431

**Published:** 2014-06-19

**Authors:** M. Sohel Rahman, M. Kaykobad, Jesun Sahariar Firoz

**Affiliations:** ^1^A*ℓ*EDA Group, Department of CSE, BUET, Dhaka 1000, Bangladesh; ^2^Department of CSE, BUET, Dhaka 1000, Bangladesh

## Abstract

A Hamiltonian path in a graph is a path involving all the vertices of the graph. In this paper, we revisit the famous Hamiltonian path problem and present new sufficient conditions for the existence of a Hamiltonian path in a graph.

## 1. Introduction

Hamiltonian paths and cycles are named after William Rowan Hamilton who invented the puzzle that involves finding a Hamiltonian cycle in the edge graph of the dodecahedron. Although Hamilton solved this particular puzzle, finding Hamiltonian cycles or paths in arbitrary graphs is proved to be among the hardest problems of computer science [1]. As a result, instead of complete characterization, most researchers aimed to find sufficient conditions for a graph to possess a Hamiltonian cycle or path. In this paper, we focus on degree based sufficient conditions for the existence of Hamiltonian paths in a graph.

To the best of our knowledge, the quest for good sufficient degree based conditions for Hamiltonian cycles or paths dates back to 1952 when Dirac presented the following theorem, where *δ*(*G*) denotes the degree of the minimum degree vertex of the graph *G*.

**Theorem 1 (see [2]).**

If *G* is a simple graph with *n* vertices, where *n* ≥ 3 and *δ*(*G*) ≥ *n*/2, then *G* contains a Hamiltonian cycle.

Later Ore in 1960 presented a highly celebrated result where a lower bound for the degree sum of nonadjacent pairs of vertices was used to force the existence of a Hamiltonian cycle. In particular, Ore proved the following theorem, where *d*_*u*_ denotes the degree of the vertex *u*.

**Theorem 2 (see [3]).**

Let *G* be a simple graph with *n* vertices and *u*, *v* distinct nonadjacent vertices of *G* with *d*_*u*_ + *d*_*v*_ ≥ *n*. Then, *G* has a Hamiltonian cycle.

A graph satisfying Ore's condition has a diameter of only two [4], where the diameter of a graph is the longest distance between two vertices. But if a sufficient condition can be derived for a graph with diameter more than two, Hamiltonian path or cycle may be found with fewer edges. With this motivation, Rahman and Kaykobad [5] proposed a sufficient condition to find a Hamiltonian Path in a graph involving the parameter *δ*(*u*, *v*), which denotes the length of the shortest path between *u* and *v*.

**Theorem 3 (see [5]).**

Let *G* = (*V*, *E*) be a connected graph with *n* vertices such that for all pairs of distinct nonadjacent vertices *u*, *v* ∈ *V* one has *d*_*u*_ + *d*_*v*_ + *δ*(*u*, *v*) ≥ *n* + 1. Then, *G* has a Hamiltonian path.

In some subsequent literature, the condition “*d*_*u*_ + *d*_*v*_ + *δ*(*u*, *v*) ≥ *n* + 1, where *u*, *v* are distinct nonadjacent vertices of a graph having *n* vertices,” is referred to as the “Rahman-Kaykobad” condition. A number of interesting results were achieved extending and using the “Rahman-Kaykobad” condition as listed below.

**Theorem 4 (see [6]).**

Let *G* be a 2-connected graph which satisfies the “Rahman-Kaykobad” condition. If *G* contains a Hamiltonian path with endpoints at distance 3, then *G* contains a Hamiltonian cycle.

**Theorem 5 (see [7]).**

Let *G* be a connected graph which satisfies the “Rahman-Kaykobad” condition. Then, either *G* contains a Hamiltonian cycle or *G* belongs to some specific classes of graphs.

**Theorem 6 (see [8]).**

Let *G* be a 2-connected graph with *n* ≥ 3 vertices. If *d*_*u*_ + *d*_*v*_ ≥ *n* − 1 for every pair of vertices *u* and *v* with *δ*(*u*, *v*) = 2, then *G* contains a Hamiltonian cycle, unless *n* is odd and *G* belongs to some specific classes of graphs.

The sufficient conditions of Theorems 4, 5, and 6 can be seen as incremental improvements over the result of Rahman and Kaykobad [5]. To the best of our knowledge, the latest and the best (so far) result of “Rahman-Kaykobad” condition was reported in [9]. In particular, in [9], the authors show that “Rahman-Kaykobad” condition is “almost” sufficient to make a graph pancyclic, where a graph is pancyclic if it contains a cycle of length *k* for 3 ≤ *k* ≤ *n*.

**Theorem 7 (see [9]).**

Let *G* be a 2-connected graph of order *n* ≥ 6, which satisfies the “Rahman-Kaykobad” condition. Then, either *G* is pancyclic or *G* belongs to some specific classes of graphs.

In [10], the authors proved the following traceability analogue of the famous Fan's condition for hamiltonicity [11].

**Theorem 8 .**

Let *G* be a connected graph of order *n*. If max⁡ {*d*(*u*), *d*(*v*)} ≥ (*n* − 1)/2 for each pair of vertices *u*, *v* ∈ *V*(*G*) at distance 2, then *G* is traceable.

By this theorem, the existence of a Hamiltonian path for each pair of vertices of only distance two is ensured. In this paper, we mention a more generalized version of Theorem 8. In particular, we present new sufficient conditions for a graph to possess a Hamiltonian path and Theorem 8 can be seen as a special case of our sufficient conditions. This time, we achieve a lower bound for the degree sum of nonadjacent pairs of vertices that is 2 lesser than Ore's condition. However, this condition cannot guarantee a Hamiltonian path for all graphs and we present such examples as well. The rest of the paper is organized as follows. In Section 2, we present some preliminary notations and results needed throughout the paper.  Section 3 presents our main results. Finally, we very briefly conclude in Section 4.

## 2. Preliminaries

**Figure 1:**
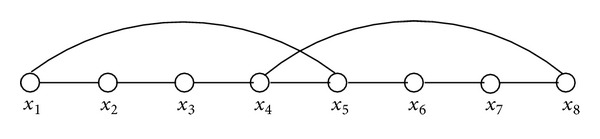
Crossover edges.

We consider only simple graphs and hence neither self-loop nor multiedges are present. Suppose we have a graph *G* = (*V*, *E*) with *n* vertices. We sometimes use the notations *V*[*G*] = *V* and *E*[*G*] = *E*. Two vertices, *u*, *v* ∈ *V* are said to be adjacent/neighbours to each other if (*u*, *v*) ∈ *E*; otherwise, they are nonadjacent. The set of neighbours of a vertex *u* in *G* is denoted by *N*_*u*_. If *G*′ is a subgraph of *G* and *u* ∈ *V*[*G*′], then *N*_*u*_[*G*′] denotes the set of neighbours of *u* (confined) in *G*′. Now, *d*_*u*_ = |*N*_*u*_| and *d*_*u*_[*G*′] = |*N*_*u*_[*G*′]|. We use $\bar{G^{\prime }}$ to denote the complimentary graph of *G*′ with respect to *G*; that is, $V\left[\bar{G^{\prime }}\right]=V-V\left[G^{\prime }\right]$ and $E\left[\bar{G^{\prime }}\right]=E-E\left[G^{\prime }\right]$. A path *P* of *G* is defined by a sequence of vertices 〈*u* = *x*_1_, *x*_2_,…, *x*_*k*_ = *v*〉 such that *V*[*P*] = {*x*_*i*_∣1 ≤ *i* ≤ *k*}⊆*V*, *E*[*P*] = {(*x*_*i*_, *x*_*i*+1_)∣1 ≤ *i* < *k*}⊆*E*. Sometimes, *P* is referred to as a *u*, *v*-path and *u* and *v* are referred to as the end vertices or endpoints of *P*. Also, sometimes we use the notation |*P*| to denote the length of *P*. So, by our definition, |*P*| = *k* − 1. If we have (*x*_1_, *x*_*k*_) ∈ *E*, then the graph *C* = (*V*[*P*], *E*′) such that *E*′ = *E*[*P*] ⋃ {(*x*_1_, *x*_*k*_)} is called a cycle. In what follows, we only consider simple paths and simple cycles. A path *P* (cycle *C*) is called a Hamiltonian path (cycle) if *V*[*P*] = *V* (*V*[*C*] = *V*). Given a path *P* of *G* as defined above, assume that *d*_*x*_1__[*P*] ≠ 0 and *d*_*x*_*k*__[*P*] ≠ 0. Two edges (*x*_1_, *x*_*i*_), (*x*_*k*_, *x*_*j*_) ∈ *E*, 1 ≤ *i* ≠ *j* ≤ *k*, are said to be crossover edges if and only if *j* = *i* − 1. For example, in Figure 1, the pair of edges (*x*_1_, *x*_5_) and (*x*_8_, *x*_4_) are crossover edges. The following is a well-known fact.

**Fact 1 .**

Suppose *P* = 〈*x*_1_, *x*_2_,…, *x*_*k*_〉 is a path of *G*. If there exist crossover edges (*x*_1_, *x*_*i*_), (*x*_*k*_, *x*_*i*−1_), then there is a cycle *C* = (*V*[*P*], *E*′) in *G*.

**Proof**

We easily get a cycle *C* as follows:

(1) $$\begin{array}{c} C=\langle x_{1},x_{2},\ldots ,x_{i-1},x_{k},x_{k-1},\ldots ,x_{i},x_{1}\rangle . \end{array}$$

In what follows, we extensively use the following result.

**Lemma 9 (see [5]).**

Let *G* = (*V*, *E*) be a connected graph with *n* vertices and *P* a longest path in *G*. If *P* is contained in a cycle then *P* is a Hamiltonian path.

An independent set of a graph *G* = (*V*, *E*) is a set of vertices *V*′⊆*V* such that all pairs of vertices *u*, *v* ∈ *V*′ are nonadjacent in *G*. A graph can be decomposed into independent sets in the sense that the entire vertex set of the graph can be partitioned into pairwise disjoint independent subsets. Such independent subsets are called partite sets or simply parts. A graph is said to be a *k*-partite graph, if its vertex set can be decomposed into *k* partite sets but not fewer. So, a bipartite graph is a graph that can be decomposed into two partite sets but not fewer. Similarly, a tripartite graph is a graph that can be decomposed into three partite sets but not fewer. A 1-partite graph is the same as an independent set or an empty graph.

One often writes *G* = (*A* ⋃ *B*, *E*) to denote a bipartite graph whose partite sets are *A* and *B*. If |*A*| = |*B*|, that is, if the two partite sets have equal cardinality, then *G* is called a balanced bipartite graph. On the other hand, if ||*A*| − |*B*|| ≤ 1, then we say that *G* is a semibalanced bipartite graph. Note that, by definition, a balanced bipartite graph is also a semibalanced bipartite graph. It is easy to see that, for a bipartite graph *G* to possess a Hamiltonian path, *G* must be semibalanced. Similar to the notation used for bipartite graphs, a tripartite graph with partite sets *A*, *B*, and *C* may be denoted by *G* = (*A* ⋃ *B* ⋃ *C*, *E*).

## 3. Sufficient Conditions

In this section we present our main results. First, we present the following useful lemma.

**Lemma 10 .**

Let *P* = 〈*x*_1_, *x*_2_,…, *x*_*p*_〉 be a longest path of *G* such that *p* ≥ 4. If *d*_*x*_1__ + *d*_*x*_*p*__ ≥ *p* − 1 + *l*, *l* ≥ 1, then there exists at least *l* crossover edges.

**Proof**

Since *P* is a longest path, (*x*_1_, *x*_*p*_) ∉ *E*(Lemma 9) and all the neighbors of *x*_1_ and *x*_*p*_ must lie within *P*. Assume that *N*(*x*_1_) = {*x*_*i*_∣(*x*_1_, *x*_*i*_) ∈ *E*} and *N* ^+^(*x*_*p*_) = {*x*_*i*+1_∣(*x*_1_, *x*_*i*_) ∈ *E*,  *i* + 1 ≤ *p*}. Now, it is clear that *x*_1_, *x*_*p*_ ∉ *N*(*x*_1_) and *x*_1_, *x*_2_ ∉ *N* ^+^(*x*_*p*_). Therefore, we must have |*N*(*x*_1_) ⋃ *N* ^+^(*x*_*p*_)| ≤ *p* − 1. Now we have the following:

(2) |N(x1)⋂N+(xp)| =|N(x1)|+|N+(xp)|−|N(x1)⋃N+(xp)| =dx1+dxp−|N(x1)⋃N+(xp)| ≥dx1+dxp−p+1 ≥p−1+l−p+1 =l.

Therefore, we have |*N*(*x*_1_) ⋂ *N* ^+^(*x*_*p*_)| ≥ *l* and we are done.

**Remark 11 .**

Note carefully that, in Lemma 10, when we talk about multiple crossover edges, they may not necessarily be disjoint.

Now, we present the following sufficient condition.

**Theorem 12 .**

Let *G* = (*V*, *E*) be a connected graph with *n* ≥ 5 vertices, such that *n* is odd. If for all pairs of nonadjacent vertices *u*, *v* one has *d*_*u*_ + *d*_*v*_ ≥ *n* − 2, then *G* has a Hamiltonian path.

**Proof**

Let *P* = 〈*x*_1_, *x*_2_,…, *x*_*k*_〉 be a longest path of *G* of length *k* − 1. If *P* is a Hamiltonian path, we are done. So, assume otherwise. Clearly, (*x*_1_, *x*_*k*_) ∉ *E* because, otherwise, by Lemma 9, we are done. Now, we must have $d_{x_{1}}\left[\bar{P}\right]=d_{x_{k}}\left[\bar{P}\right]=0$, since, otherwise, *P* is part of a longer path, a contradiction. Therefore, we must have *d*_*x*_1__ = *d*_*x*_1__[*P*] and *d*_*x*_*k*__ = *d*_*x*_*k*__[*P*].

**Figure 2:**
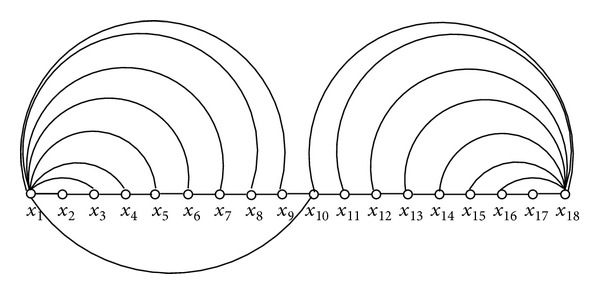
Config-1.

**Figure 3:**
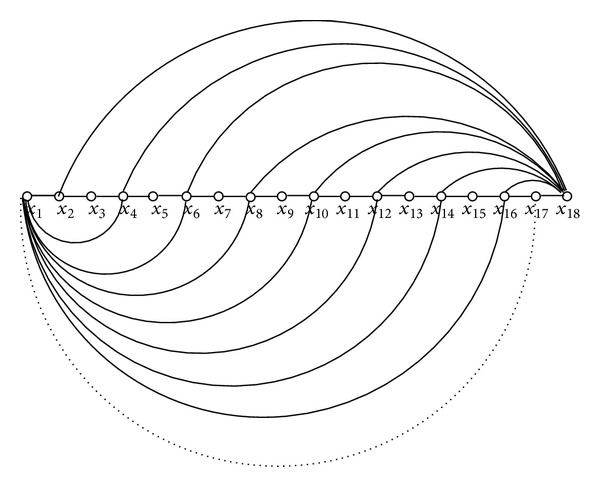
Config-2: *k* is even (the dotted edge is nonexistent).

**Figure 4:**
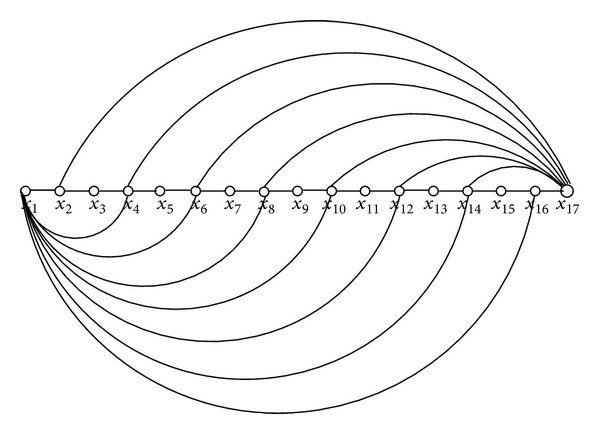
Config-2: *k* is odd.

**Figure 5:**
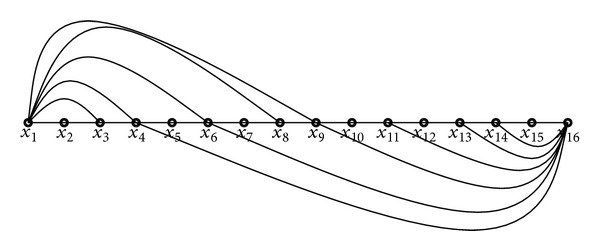
Config-3: a combination of Config-1 and Config-2.

Now, since *x*_1_, *x*_*k*_ are nonadjacent, we must have *d*_*x*_1__ + *d*_*x*_*k*__ ≥ *n* − 2. Now, if we assume that *d*_*x*_1__ + *d*_*x*_*k*__ < *k* − 1, then we must have *k* − 2 ≥ *n* − 2; that is, *k* ≥ *n*, implying that *P* is a Hamiltonian path. Therefore, assume that *d*_*x*_1__ + *d*_*x*_*k*__ ≥ *k* − 1. Since we cannot allow a crossover edge, if we want to maximize the total degree sum of *d*_*x*_1__ + *d*_*x*_*k*__, we can have configurations similar to one of three configurations, namely, Config-1, Config-2, and Config-3 shown in Figures 2 to 5. It can be verified easily that any other configuration will result in *d*_*x*_1__ + *d*_*x*_*k*__ < *k* − 1 and according to the argument presented above *P* then would have to be a Hamiltonian path. Note carefully that in both Config-1 and Config-2 we have *d*_*x*_1__ + *d*_*x*_*k*__ ≥ *k* − 1. The main properties of Config-1 and Config-2 are listed as the following facts. And Config-3 is a combination of Config-1 and Config-2.

*Fact 2*. In Config-1, we have *N*_*x*_1__ = {*x*_*j*_∣1 < *j* ≤ *r*}, *N*_*x*_*k*__ = {*x*_*j*_∣*r* ≤ *j* < *k*}, such that *N*_*x*_1__ ⋂ *N*_*x*_*k*__ = {*x*_*r*_}.

*Fact 3*. In Config-2, if *k* is odd, then we have *N*_*x*_1__ = *N*_*x*_*k*__ = {*x*_*j*_∣*j*  is  even}. On the other hand, if *k* is even, then we have *N*_*x*_1__ = {*x*_*j*_∣*j*  is  even  and  *j* ≠ *k*} and *N*_*x*_*k*__ = *N*_*x*_1__ ⋃ {*x*_*k*−1_}.

Now, we consider two cases as follows.

*Case 1*  (*d*_*x*_1__ + *d*_*x*_*k*__ > *k* − 1). From Figures 2 to 4 it is easy to see that if *d*_*x*_1__ + *d*_*x*_*k*__ ≥ *k*, then we definitely will have a crossover edge resulting in a cycle containing another path *P*′ such that |*P*| = |*P*′|. Therefore, by Lemma 9, it follows that the length of a longest path cannot be *k* − 1, a contradiction.

*Case 2*  (*d*_*x*_1__ + *d*_*x*_*k*__ = *k* − 1). Since (*x*_1_, *x*_*k*_) ∉ *E*, we have *d*_*x*_1__ + *d*_*x*_*k*__ ≥ *n* − 2. Therefore, *k* − 1 ≥ *n* − 2⇒*k* ≥ *n* − 1. Now, if *k* > *n* − 1, we are done. So, assume that *k* = *n* − 1. Then we have a vertex *y* such that *y* ∈ *V* − *V*[*P*]. Now, for *y*, we cannot have (*y*, *x*_1_), (*y*, *x*_*k*_) ∈ *E* because, then, we have a longer path than *P*, a contradiction. Now we must have *d*_*y*_ + *d*_*x*_1__ ≥ *n* − 2 and *d*_*y*_ + *d*_*x*_*k*__ ≥ *n* − 2. Then, we have the following:

(3) dx1+dxk+2×dy≥2×(n−2)  ⟹2×dy+k−1≥2n−4  ⟹2×dy+n−1−1≥2n−4  ⟹2×dy+n≥2n−2  ⟹2×dy≥n−2  ⟹dy≥(n−2)2  ⟹dy≥n2−1.

Now, since *n* ≥ 5 and *d*_*y*_ cannot be a fractional value, we must have *d*_*y*_ ≥ 2. Now we have two cases.

*Case 2.a (Config-1)*. In this case we have a configuration similar to Figure 2. Now, let *x*_*i*_, *x*_*j*_ ∈ *N*_*y*_. Assume without loss of generality that *j* > *i*. If *j* = *i* + 1, then we easily get a Hamiltonian path *P*′ = 〈*x*_1_, *x*_2_,…, *x*_*i*_, *y*, *x*_*j*_, *x*_*j*+1_,…, *x*_*k*_〉 and we are done. So, assume that *j* = *i* + *l*, *l* > 1.

Now, recall that, in this case, there exists a vertex *x*_*r*_, *r* ∉ {1, *k*} such that (*x*_*r*_, *x*_1_), (*x*_*r*_, *x*_*k*_) ∈ *E* (Fact 2). Now, we have three subcases.

*Case 2.a.1 *(*r* ≤ *i* < *j* < *k*). From Fact 2, it is clear that (*x*_*k*_, *x*_*i*+1_) ∈ *E*. Therefore, we get a Hamiltonian path

(4) $$\begin{array}{c} P^{\prime \prime }=\langle x_{1},x_{2},\ldots ,x_{i},y,x_{j},x_{j+1},\ldots ,x_{k},x_{i+1},x_{i+2},\ldots ,x_{j-1}\rangle \end{array}$$

and we are done.

*Case 2.a.2 *(1 < *i* < *j* ≤ *r*). This is symmetrical to Case 2.a.1.

*Case 2.a.3 *(1 < *i* < *r* < *j*). Again, from Fact 2, it is clear that (*x*_*k*_, *x*_*j*−1_) ∈ *E*. Therefore, we get a Hamiltonian path

(5) $$\begin{array}{c} P^{\prime \prime \prime }=\langle x_{1},x_{2},\ldots ,x_{i},y,x_{j},x_{j+1},\ldots ,x_{k},x_{j-1},x_{j-2},\ldots ,x_{i+1}\rangle , \end{array}$$

and we are done.

*Case 2.b (Config-2)*. Since *n* is odd, in this case, we have *k* is even. Hence, we have a configuration similar to Figure 3. Recall that we have *d*_*y*_ ≥ (*n*/2) − 1. Since *n* is odd and *d*_*y*_ cannot be a fractional number, we must have *d*_*y*_ ≥ ((*n* + 1)/2) − 1. In other words, we have *d*_*y*_ ≥ ((*k* + 2)/2) − 1 = *k*/2.

First of all, if we have either of the edges (*y*, *x*_1_), (*y*, *x*_*k*_) ∈ *E*, we are done. So assume otherwise. Furthermore, if we have (*y*, *x*_*k*−1_) ∈ *E*, then we get a Hamiltonian path *P*′ = 〈*x*_1_, *x*_2_,…, *x*_*k*−2_, *x*_*k*_, *x*_*k*−1_, *y*〉 and we are done. So, assume otherwise. So, *x*_1_, *x*_*k*−1_, *x*_*k*_ ∉ *N*_*y*_. Therefore, we have *k* − 3 vertices as candidates for membership in *N*_*y*_.

Now, since *n* ≥ 5, we have *k* = *n* − 1 ≥ 4. Therefore, we must have *d*_*y*_ ≥ 2. Now, let *x*_*i*_, *x*_*j*_ ∈ *N*_*y*_. Without the loss of generality assume that *j* > *i*. Clearly, if *j* = *i* + 1, we are done, since we get a Hamiltonian path *P*′ = 〈*x*_1_, *x*_2_,…, *x*_*i*_, *y*, *x*_*j*_, *x*_*j*+1_,…, *x*_*k*_〉. So, assume that *j* = *i* + *l*, *l* > 1. Since *k* − 3 is an odd number, it follows that *d*_*y*_ ≤ (*k* − 2)/2 = (*k*/2) − 1. This contradicts our deduction above that *d*_*y*_ ≥ *k*/2.

*Case 2.c (Config-3)*. As mentioned above Config-3 is a combination of Config-1 and Config-2. Recall that in this case our assumption is *d*_*x*_1__ + *d*_*x*_*k*__ = *k* − 1. Hence we have *d*_*y*_ ≥ (*n*/2) − 1. Clearly, *x*_1_, *x*_*k*_ ∉ *N*_*y*_ because then we get a longer path, a contradiction. It is easy to verify that this would force two consecutive vertices *x*_*i*_, *x*_*i*+1_ ∈ *N*_*y*_ for 2 ≤ *i* ≤ *n* − 2. Then, again we get a longer path simply including the subpath 〈*x*_*i*_, *y*, *x*_*i*+1_〉, which leads us to a contradiction.

And this completes our proof.

Note that in the proof of Theorem 12, the condition that *n* is odd is assumed only in Case 2.b where Config-2 is considered. So, based on the proof of Theorem 12, we have the following two corollaries.

**Corollary 13 .**

Suppose one has a graph *G* = (*V*, *E*) with *n* ≥ 5 vertices such that for all pairs of nonadjacent vertices *u*, *v* one has *d*_*u*_ + *d*_*v*_ ≥ *n* − 2. If one has configurations similar to Config-1, then *G* must possess a Hamiltonian path.

**Corollary 14 .**

Suppose one has a graph *G* = (*V*, *E*) with *n* ≥ 5 vertices such that for all pairs of nonadjacent vertices *u*, *v* one has *d*_*u*_ + *d*_*v*_ ≥ *n* − 2. If one has configurations similar to Config-2 and *n* is odd, then *G* must possess a Hamiltonian path.

We now present another sufficient condition.

**Theorem 15 .**

Let *G* = (*A* ⋃ *B*, *E*) be a semibalanced bipartite connected graph with *n* ≥ 5 vertices. If, for all pairs of nonadjacent vertices *u*, *v* one has *d*_*u*_ + *d*_*v*_ ≥ *n* − 2, then *G* has a Hamiltonian path.

*Proof*. Now, the proof assumes the same hypotheses of the proof of Theorem 12. Clearly, based on Corollaries 13 and 14, it suffices to consider only Config-2 when *n* is even. Therefore, what follows should be treated as a continuation of the proof of Theorem 12 excluding Case 2.b. Additionally, we assume that *G* is a semibalanced bipartite connected graph.

Since *n* is even, in this case, *k* is odd. Hence, we have a configuration similar to Figure 4. Recall that we have *d*_*y*_ ≥ (*n*/2) − 1. In other words, we have *d*_*y*_ ≥ ((*k* + 1)/2) − 1 = (*k*/2)+(1/2) − 1 = (*k*/2)−(1/2) = (*k* − 1)/2.

First of all, if we have either of the edges (*y*, *x*_1_), (*y*, *x*_*k*_) ∈ *E*, we are done. So assume otherwise. So, *x*_1_, *x*_*k*_ ∉ *N*_*y*_. Therefore, we have *k* − 2 vertices as candidates for membership in *N*_*y*_.

Now, since *n* is even, *n* ≥ 5 implies *n* ≥ 6. Hence, we have *k* = *n* − 1 ≥ 5. Therefore, we must have *d*_*y*_ ≥ 2. Now, let *x*_*i*_, *x*_*j*_ ∈ *N*_*y*_. Without the loss of generality assume that *j* > *i*. Clearly, if *j* = *i* + 1, we are done, since we get a Hamiltonian path *P*′ = 〈*x*_1_, *x*_2_,…, *x*_*i*_, *y*, *x*_*j*_, *x*_*j*+1_,…, *x*_*k*_〉. So, assume that *j* = *i* + *l*, *l* > 1. Now, we claim the following.

**Claim 1 .**

If *x*_*i*_, *x*_*j*_ ∈ *N*_*y*_ such that either *i* or *j* or both are odd, then we have a Hamiltonian path.

**Proof**

Assume that *i* is even and *j* = *i* + *l*, *l* is odd. Clearly, *j* − 1 is even and we know that *x*_*j*−1_ is adjacent to both *x*_1_ and *x*_*k*_ (Fact 3). Then, we easily get a Hamiltonian path

(6) $$\begin{array}{c} P^{\prime \prime }=\langle x_{1},x_{2},\ldots ,x_{i},y,x_{j},x_{j+1},\ldots ,x_{k},x_{j-1},x_{j-2},\ldots x_{i+1}\rangle . \end{array}$$

Similarly we can show the existence of another Hamiltonian path if *i* is odd and *j* is even. The case when both *i* and *j* are odd also follows easily.

Now, by Claim 1, if *x*_*i*_, *x*_*j*_ ∈ *N*_*y*_, such that either *i* or *j* or both are odd, then we have a Hamiltonian path and we are done. Therefore, assume otherwise. Then, since we have *k* − 2 vertices as candidates for membership in *N*_*y*_, *d*_*y*_ ≥ (*k* − 1)/2, and *k* is odd, we must have *N*_*y*_ = {*x*_*j*_∣*j*  is  even}. Therefore, *d*_*y*_ = (*k* − 1)/2.

Now, if *i*, *j* are odd, then we must have (*x*_*i*_, *x*_*j*_) ∉ *E*, because otherwise we get an odd cycle contradicting our assumption that *G* is a bipartite graph. Similarly, if *i*, *j* are even, then we must have (*x*_*i*_, *x*_*j*_) ∉ *E* for the same reason. Then, the two partite sets *A* and *B* of our graph *G* = (*A* ⋃ *B*, *E*) are defined as follows:

(7) $$\begin{array}{c} A=\left\{x_{i}\;|\;i\;\text{is}\;\text{even}\right\},\\ B=\left\{x_{i}\;|\;i\;\text{is}\;\text{odd}\right\}⋃\left\{y\right\}. \end{array}$$

Then, we have |*B*| − |*A*| > 1, which contradicts our assumption that *G* is semibalanced and this completes the proof of Theorem 15.

Interestingly, we have the following theorem as well.

**Theorem 16 .**

Let *G* = (*A* ⋃ *B* ⋃ *C*, *E*) be a tripartite connected graph with *n* ≥ 5 vertices. If, for all pairs of nonadjacent vertices *u*, *v* one has *d*_*u*_ + *d*_*v*_ ≥ *n* − 2, then *G* has a Hamiltonian path.

**Proof**

The proof of this theorem almost exactly follows the proof of Theorem 15. The only difference now is that we assume *G* to be tripartite instead of semibalanced bipartite. Then, we can continue with the same arguments as we did in the proof of Theorem 15 and reach a position where *G* turns out to be a bipartite graph. This is a contradiction since *G* is tripartite and this completes the proof.

### 3.1. Discussion

**Figure 6:**
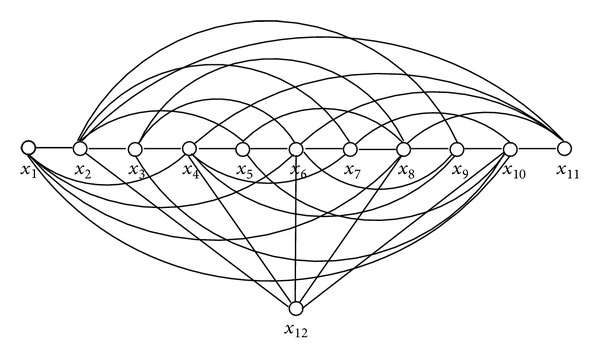
A (problematic) graph with even number of vertices.

In Theorem 12, we have presented a sufficient condition for the existence of Hamiltonian path assuming that, *n*, the number of vertices of the graph, is odd. When *n* is even, we have shown our condition to be effective for some classes of graphs. Interestingly, we can construct a graph with even number of vertices which satisfies our condition but still does not possess a Hamiltonian path. For example, consider the graph in Figure 6. The adjacency matrix of the graph of Figure 6 is presented in Table 1. Now, it is clear from Table 1 that for every pair of nonadjacent vertices *u*, *v* in this graph we have *d*_*u*_ + *d*_*v*_ ≥ 10 = *n* − 2. However, the graph in Figure 6 is in fact a bipartite graph with partite sets *A* = {*x*_1_, *x*_3_, *x*_5_, *x*_7_, *x*_9_, *x*_11_, *x*_12_} and *B* = {*x*_2_, *x*_4_, *x*_6_, *x*_8_, *x*_10_} such that |*A* | −|*B* | >1. Therefore, it cannot possess a Hamiltonian path. Also, note that even if we make the graph nonbipartite by adding some edges in the partite set *B*, still the graph will not have a Hamiltonian path. In fact, even if we add all the edges to make the partite set *B* a clique, the graph will not possess a Hamiltonian path. On the other hand, a single edge within the partite set *A* can provide us a Hamiltonian path.

## 4. Conclusion

In this paper, we have presented new degree based sufficient conditions for a graph to contain a Hamiltonian path. It would be interesting to investigate whether our condition could force a graph to contain a Hamiltonian cycle or even to be pancyclic.

## Figures and Tables

**Table 1:** Adjacency matrix of the graph of Figure 6.

	*x*_1_	*x*_2_	*x*_3_	*x*_4_	*x*_5_	*x*_6_	*x*_7_	*x*_8_	*x*_9_	*x*_10_	*x*_11_	*x*_12_
*x*_1_	—	1	0	1	0	1	0	1	0	1	0	0
*x*_2_	1	—	1	0	1	0	1	0	1	0	1	1
*x*_3_	0	1	—	1	0	1	0	1	0	1	0	0
*x*_4_	1	0	1	—	1	0	1	0	1	0	1	1
*x*_5_	0	1	0	1	—	1	0	1	0	1	0	0
*x*_6_	1	0	1	0	1	—	1	0	1	0	1	1
*x*_7_	0	1	0	1	0	1	—	1	0	1	0	0
*x*_8_	1	0	1	0	1	0	1	—	1	0	1	1
*x*_9_	0	1	0	1	0	1	0	1	—	1	0	0
*x*_10_	1	0	1	0	1	0	1	0	1	—	1	1
*x*_11_	0	1	0	1	0	1	0	1	0	1	—	0
*x*_12_	0	1	0	1	0	1	0	1	0	1	0	—

Degree	5	7	5	7	5	7	5	7	5	7	5	5
